# Erratum: Cooling a mechanical resonator with nitrogen-vacancy centres using a room temperature excited state spin–strain interaction

**DOI:** 10.1038/ncomms16166

**Published:** 2017-11-29

**Authors:** E. R. MacQuarrie, M. Otten, S. K. Gray, G. D. Fuchs

Nature Communications 8: Article number: 14358; DOI: 10.1038/ncomms14358 (2017); Published 02 06 2017; Updated 11 29 2017.

This Article contains a typographical error in the sentence preceding Eq. 4. This sentence should read, ‘Under the secular approximation and working in the limit 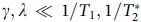, the dynamical equation for the phonon occupancy 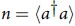 can be simplified to’; that is, with a much-less-than symbol rather than a much-greater-than symbol.

There is a further error in Fig 4. The label on the upper state of the right-most transition in Fig. 4b should be “
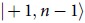
” not “
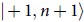
”.

