# Supplementary material for: Cooling a mechanical resonator with nitrogen-vacancy centres using a room temperature excited state spin–strain interaction
Source: Nat Commun. 2017 Feb 6;8:14358. doi: 10.1038/ncomms14358 (PMC5303879; doi:10.1038/ncomms14358)
Supplement: Supplementary Information — Supplementary Figures, Supplementary Table 1, Supplementary Notes and Supplementary References [file ncomms14358-s1.pdf]

SUPPLEMENTARY NOTE 1: SIGN OF $A_{\parallel}^{\mathrm{e}}$

To confirm the reversal in the sign of the hyperfine coupling $A_{\parallel}$ between the ground state (GS) and excited state (ES) orbitals, we mechanically drive spin population in the ES conditional on the spin state of the ^{14}N nucleus as established within the GS orbital. These measurements follow the pulse sequence depicted in Supplementary Fig. 1a. This modified pulse sequence replaces the hard π-pulses used to quantify $d_{\perp}^{\mathrm{e}}$ in the main text with weak π-pulses conditional on the nuclear spin state.

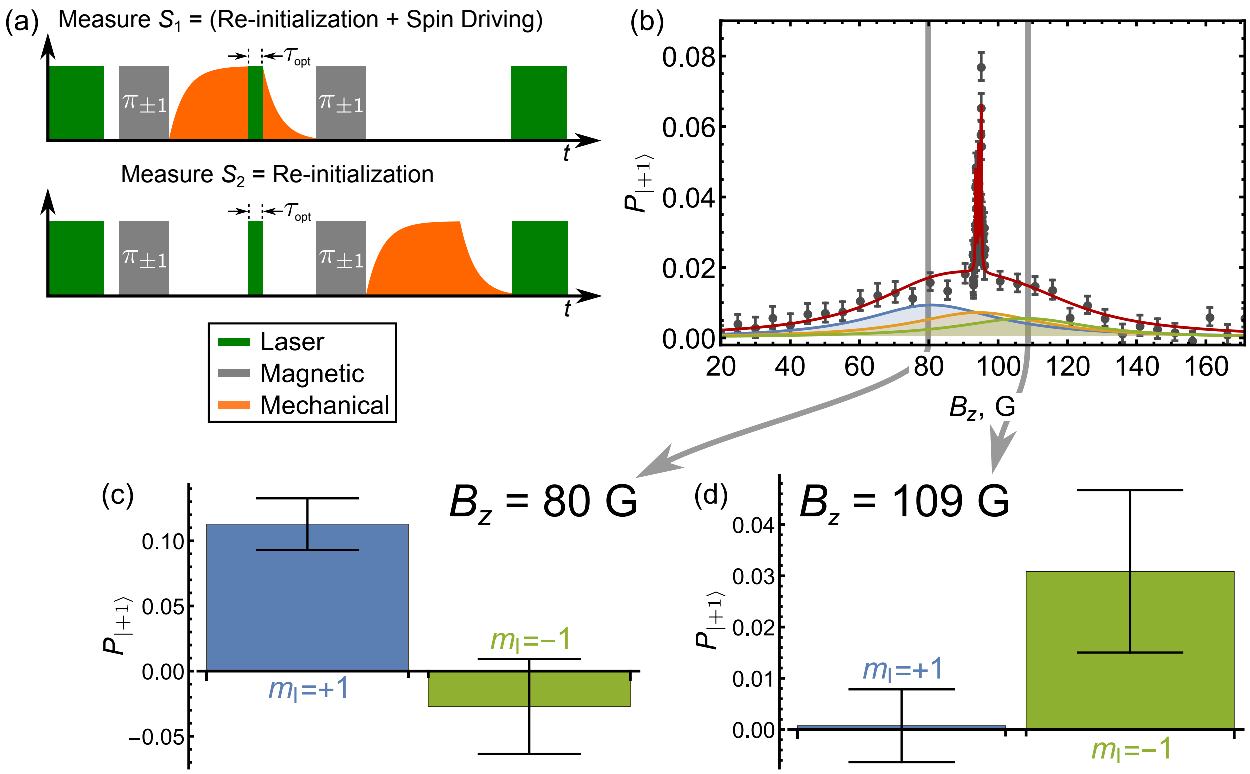

Supplementary Figure 1: Measuring the sign of $A_{\parallel}^{\mathrm{e}}$. (a) Pulse sequence used to verify the sign of $A_{\parallel}^{\mathrm{e}}$. (b) Mechanically driven spin contrast as a function of B_z. The red curve is a least squares fit to the sum of six Lorentzians. (c,d) Measurements taken at the (c) low-field and (d) high-field hyperfine resonances that were conditional on the ^{14}N nuclear spin state. The data in (b-d) were measured on a single device with an NV center ensemble, and error bars are from the standard deviation in photon counting.

As shown in Supplementary Fig. 1b, we perform this measurement at the high-field and low-field ES hyperfine resonances. We observe mechanically-driven spin population in the

$m_\mathrm{I} = +1$ manifold at the $B_z = 80$ G resonance and in the $m_\mathrm{I} = -1$ manifold at the $B_z = 109$ G resonance. The resonance condition for spin driving is $\omega_\mathrm{m} = 2\gamma_\mathrm{NV} B_z + 2A^\mathrm{e}_\parallel m_\mathrm{I}$, giving $A^\mathrm{e}_\parallel = \left(\frac{1}{2}\omega_\mathrm{m} - \gamma_\mathrm{NV} B_z\right)/m_\mathrm{I}$. Using the parameter values given in the main text, this gives $A^\mathrm{e}_\parallel/2\pi = +40$ MHz and confirms the sign of $A^\mathrm{e}_\parallel$.

SUPPLEMENTARY NOTE 2: GROUND STATE MECHANICALLY DRIVEN RABI OSCILLATIONS

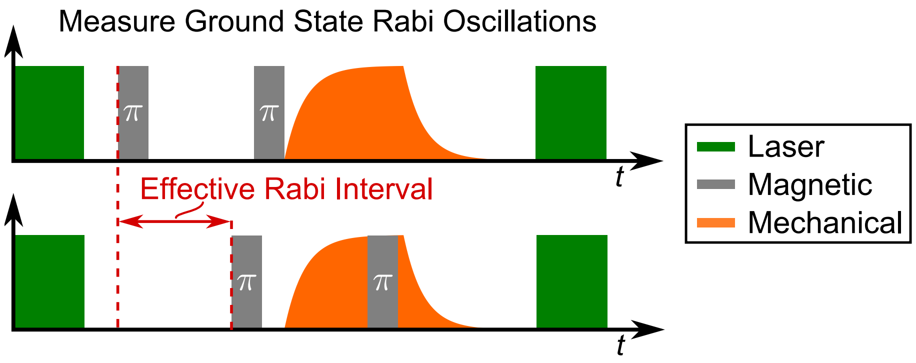

Supplementary Figure 2: Ground state mechanical Rabi driving. Pulse sequence used to measure mechanically driven ground state Rabi oscillations.

The GS mechanically driven Rabi oscillations used to quantify Ω_g were measured using the pulse sequence shown in Supplementary Fig. 2. As described in detail in Supplementary Ref. [1], varying the pulse length of our mechanical driving field introduces bandwidth-related artifacts to a Rabi measurement. Instead, we fix the length of the mechanical pulse and vary the interaction time by sweeping a pair of magnetic π-pulses through the mechanical pulse. This yields the data seen in Fig. 3a of the main text where the "Effective Rabi Interval" label on the x-axis corresponds to the delay of the π-pulse pair.

For a single NV center, a GS Rabi measurement is described by the function

$$P_{|+1\rangle}(t, \Omega_\mathrm{g}) = \frac{1}{2}\left\{1 - e^{-t/T_\mathrm{Rabi}} \cos[\Omega_\mathrm{g} \tau(t)]\right\} \tag{1}$$

where $\tau(t) = \int_0^t \left(1 - e^{-t'/\tau_Q}\right) dt' = \left[\left(e^{-t/\tau_Q} - 1\right)\tau_Q + t\right]$ accounts for the ring-up of the mechanical resonator and $\tau_Q = 2Q/\omega_0$. For an ensemble measurement, we account for spatial inhomogeneities by taking the weighted average of Supplementary Eq. 1 over the

PSF of our microscope:

$$P^{\text{ens}}_{|+1\rangle} = \frac{\int_0^\infty P_{|+1\rangle}(t, \Omega_{\text{g}} |\sin[2\pi z/\lambda]|)\Gamma_{\text{opt}}(z)dz}{\int_0^\infty \Gamma_{\text{opt}}(z)dz}. \tag{2}$$

Here, we approximate the PSF by the function

$$\Gamma_{\text{opt}}(z) = \Gamma_0 \left\{ \frac{\sin\{\kappa[z_0](z - z_0)\}}{\kappa[z_0](z - z_0)} \right\}^2 \tag{3}$$

where Γ_0 is the peak optical pumping rate, $\kappa[z_0]$ defines the depth-dependent PSF width [2], z is the distance below the diamond surface, and $z_0 = 7.9 \pm 0.9$ μm is the focus depth of the PSF. We discretize the integral in Supplementary Eq. 2 and fit each GS Rabi curve, fixing τ_Q to be the same across the fits.

SUPPLEMENTARY NOTE 3: STEADY STATE PHONON OCCUPANCY

Within the two-level toy model used to analyze the proposed cooling protocol, the spin-resonator dynamics for a single NV center coupled to a mechanical resonator are governed by

$$\dot{\rho} = -i[H, \rho] + \mathcal{L}_\Gamma \rho + \mathcal{L}_\gamma \rho \tag{4}$$

where H, $\mathcal{L}_\Gamma \rho$, and $\mathcal{L}_\gamma \rho$ are defined in the main text. Using these expressions and the ladder operator commutation relations, we derive the system of ordinary differential equations that

governs the time evolution of the second order moments [3]. This system is given by

$$\frac{d}{dt}\langle a^\dagger a\rangle = (-i\lambda)\left(\langle S_+a^\dagger\rangle - \langle S_-a\rangle - \langle S_+a\rangle + \langle S_-a^\dagger\rangle\right) + \gamma n_{\mathrm{th}} - \gamma\langle a^\dagger a\rangle,$$
$$\frac{d}{dt}\langle S_+S_-\rangle = (-i\lambda)\left(\langle S_+a^\dagger\rangle - \langle S_-a\rangle - \langle S_-a^\dagger\rangle + \langle S_+a\rangle\right) - \Gamma_\perp\langle S_+S_-\rangle,$$
$$\frac{d}{dt}\langle S_+a^\dagger\rangle = (i\lambda)\left(1 + \langle a^\dagger a^\dagger\rangle + \langle S_+S_+\rangle + \langle S_+S_-\rangle + \langle a^\dagger a\rangle\right) - \left(\frac{1}{2}\Gamma_\perp + \frac{1}{2}\gamma - 2i\omega_{\mathrm{m}} + \Gamma_\parallel\right)\langle S_+a^\dagger\rangle,$$
$$\frac{d}{dt}\langle S_-a^\dagger\rangle = (-i\lambda)\left(\langle a^\dagger a\rangle + \langle a^\dagger a^\dagger\rangle - \langle S_+S_-\rangle - \langle S_-S_-\rangle\right) - \left(\frac{1}{2}\Gamma_\perp + \frac{1}{2}\gamma + \Gamma_\parallel\right)\langle S_-a^\dagger\rangle,$$
$$\frac{d}{dt}\langle S_+a\rangle = (i\lambda)\left(\langle a^\dagger a\rangle + \langle aa\rangle - \langle S_+S_-\rangle - \langle S_+S_+\rangle\right) - \left(\frac{1}{2}\Gamma_\perp + \frac{1}{2}\gamma + \Gamma_\parallel\right)\langle S_+a\rangle,$$
$$\frac{d}{dt}\langle S_-a\rangle = (-i\lambda)\left(1 + \langle aa\rangle + \langle S_-S_-\rangle + \langle S_+S_-\rangle + \langle a^\dagger a\rangle\right) - \left(\frac{1}{2}\Gamma_\perp + \frac{1}{2}\gamma + 2i\omega_{\mathrm{m}} + \Gamma_\parallel\right)\langle S_-a\rangle,$$
$$\frac{d}{dt}\langle S_-S_-\rangle = (-2i\lambda)\left(\langle S_-a^\dagger\rangle + \langle S_-a\rangle\right) - \left(\Gamma_\perp + 2i\omega_{\mathrm{m}} + \frac{1}{2}\Gamma_\parallel\right)\langle S_-S_-\rangle,$$
$$\frac{d}{dt}\langle S_+S_+\rangle = (2i\lambda)\left(\langle S_+a^\dagger\rangle + \langle S_+a\rangle\right) - \left(\Gamma_\perp - 2i\omega_{\mathrm{m}} + \frac{1}{2}\Gamma_\parallel\right)\langle S_+S_+\rangle,$$
$$\frac{d}{dt}\langle a^\dagger a^\dagger\rangle = (2i\lambda)\left(\langle S_+a^\dagger\rangle + \langle S_-a^\dagger\rangle\right) - (\gamma - 2i\omega_{\mathrm{m}})\langle a^\dagger a^\dagger\rangle,$$

and

$$\frac{d}{dt}\langle aa\rangle = (-2i\lambda)\left(\langle S_+a\rangle + \langle S_-a\rangle\right) - (\gamma + 2i\omega_{\mathrm{m}})\langle aa\rangle \tag{5}$$

where $\Gamma_\parallel = 1/T^*_{2\mathrm{e}}$, $\Gamma_\perp = 1/T_{1\mathrm{e}}$, and the other parameters are as defined in the main text.

We make the secular approximation of ignoring the fast-oscillating double-raising and double-lowering terms and solve this system in the limit $\gamma, \lambda \ll \Gamma_\perp, \Gamma_\parallel$. The time evolution of the phonon occupancy $n = \langle a^\dagger a\rangle$ can then be rewritten

$$\frac{dn}{dt} = \gamma\left(n_{\mathrm{th}} - n\right) - \frac{4\lambda^2}{2\Gamma_\parallel + \Gamma_\perp}n \tag{6}$$

as quoted in the main text.

SUPPLEMENTARY NOTE 4: CONTROL FIELDS FOR COOLING

The proposed cooling protocol requires a static magnetic bias field B_z to bring the spin-strain interaction into resonance, a continuous gigahertz-frequency magnetic field Ω_{mag} to address the $|g,0\rangle \leftrightarrow |g,-1\rangle$ transition, and continuous optical illumination Γ_{opt} to re-initialize the system.

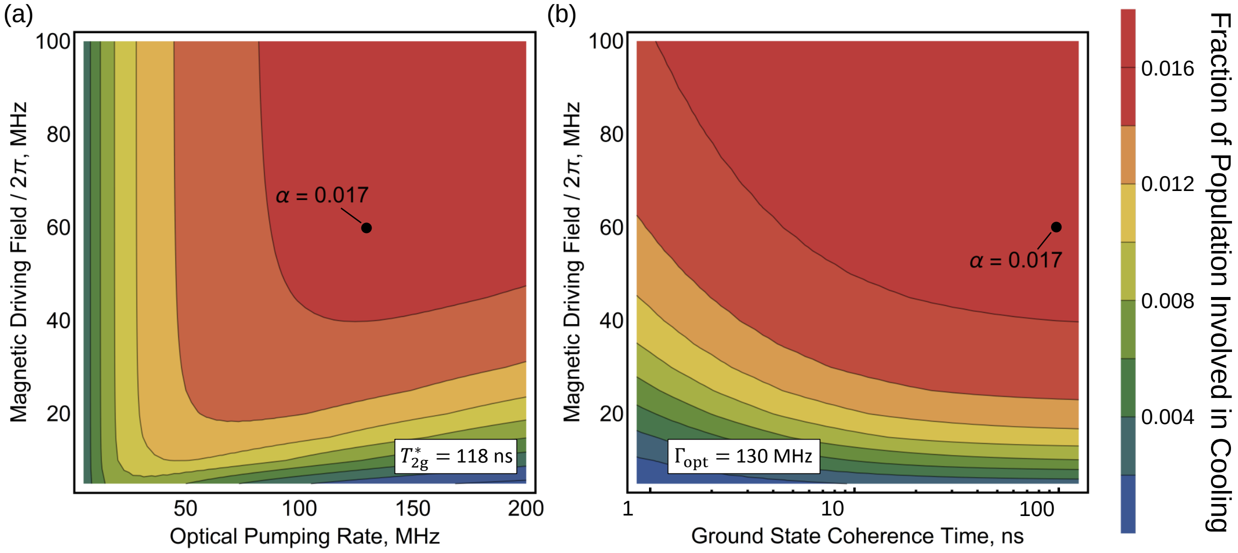

Supplementary Figure 3: Control fields for resonator cooling. The fraction α of the ensemble population involved in the cooling plotted as a function of the magnetic driving field Ω_{mag} and (a) the optical pumping rate Γ_{opt} or (b) the ground state coherence time $T_{2\mathrm{g}}^*$.

For the $\omega_0/2\pi = 1$ GHz resonators considered in this work, $B_z = \left(\omega_{\mathrm{m}}/2 - A_{\|}\right)/\gamma_{\mathrm{NV}} = 160$ G. The magnitudes of Ω_{mag} and Γ_{opt} determine α, the fraction of the ensemble population involved in the cooling. A large Γ_{opt} is desired to saturate the steady state population in the NV center ES. A large Ω_{mag} is also required to maximize the spin population driven into $|g, -1\rangle$. As shown in Supplementary Fig. 3a, α saturates for large control fields at $\alpha \sim 0.017$. Here, we have used a GS coherence time of $T_{2\mathrm{g}}^* = 118$ ns as reported in Supplementary Ref. [4] for an NV center ensemble with $\nu = 7 \times 10^{17}$ cm^{-3}.

This low value of α reduces the cooling performance of the proposed protocol and can be understood intuitively by comparing the decay rate from the ES to the GS through the metastable state $|S_1\rangle$ k_{esg} to the rate of decay directly to the GS k_{eg}. Using the rates quoted in Table 1 of the main text, this gives the ratio $k_{\mathrm{esg}}/k_{\mathrm{eg}} \sim 0.02$, which is comparable to $\alpha \sim 0.017$ and suggests that most of the spin population has become trapped in $|S_1\rangle$. Examining the steady state density matrix used to calculate α, we see that this is indeed the case, and $|S_1\rangle$ contains $\sim 73\%$ of the steady state spin population. Without a means of selectively depopulating $|S_1\rangle$, the average fraction of the ensemble involved in the cooling is

thus maximized at $\alpha \sim 0.017$.

For the analysis of our cooling protocol presented in the main text, we use $\Omega_{\rm mag}/2\pi =$ 60 MHz and $\Gamma_{\rm opt} = 130$ MHz, which gives $\alpha = 0.017$. The large $\Omega_{\rm mag}$ required to achieve this α has been previously demonstrated in ground state spin control experiments [5], and NV centers are regularly optically pumped to saturation. The scale of these control fields is therefore experimentally reasonable. As demonstrated by Supplementary Fig. 3b, for $\Omega_{\rm mag}/2\pi = 60$ MHz and $\Gamma_{\rm opt} = 130$ MHz, α remains robust until $T_{\rm 2g}^*$ becomes comparable to $T_{\rm 2e}^* = 6$ ns.

SUPPLEMENTARY NOTE 5: HIGHER ORDER MECHANICAL MODES

The eigenfrequencies of the mechanical modes scale with the resonator dimensions as $\omega_n = \kappa_n t/l^2$. For a beam, $\kappa_n = 120, 330, 628, ...$ GHz·µm. We limit our analysis to $\omega_0/2\pi =$ 1 GHz resonators. The next order mode of such a resonator will be $\omega_1/2\pi = 2.8$ GHz. Assuming a transform-limited ES linewidth of $\sim 1/T_{\rm 2e}^* = 170$ MHz, the resulting spectral isolation is more than enough to isolate the spin dynamics from higher order mechanical modes.

SUPPLEMENTARY NOTE 6: VALIDATION OF TWO-LEVEL MODEL

As the main text explains, to study our proposed cooling protocol we simplify the full seven-level NV center to an effective two-level spin system. To validate our use of this two-state distillation (TSD), we compare the results of the TSD analysis to the cooling predicted a Lamb-Dicke (LD) treatment of the seven-level NV model and to numerical simulations of the full seven-level NV center-plus-resonator Hamiltonian. Our simplification has two potential sources of error: the reduction from the seven-level system to the two-level system for one NV and the scaling from one NV to many NVs.

The LD treatment offers a powerful route to calculating $n_{\rm f}$ when a dissipative, multi-level system such as an NV center spin is coupled to a mechanical resonator and the multi-level system's dynamics are much faster than the resonator dynamics. Under the LD approximation, the NV center spin can be adiabatically eliminated from the resonator dynamical equation, and the cooling rate from a single NV center is given by $\Gamma_{{\rm c},i} =$

$2\lambda^2 \{\mathrm{Re}\,[S(\omega_\mathrm{m})] - \mathrm{Re}\,[S(-\omega_\mathrm{m})]\}$. Here, $S(\omega)$ is the spectral function of the ES spin-strain interaction, which we calculate as described in Supplementary Refs. [6] and [7] for analogous systems. For an ensemble of N spins, the total cooling rate is the sum of the individual rates, giving $\Gamma_\mathrm{c} = 2\{\mathrm{Re}\,[S(\omega_\mathrm{m})] - \mathrm{Re}\,[S(-\omega_\mathrm{m})]\} \sum_{i=1}^{N} \lambda_i^2$. This allows us to define the effective ensemble-resonator coupling $\lambda_\mathrm{eff} = \sqrt{\sum_{i=1}^{N} \lambda_i^2}$, just as we do in the TSD. In the limit $\gamma n_\mathrm{th}, \lambda_\mathrm{eff}\sqrt{\langle n\rangle + 1/2} \ll 1/T_{2\mathrm{e}}^*, 1/T_{1\mathrm{e}}, \omega_\mathrm{m}$, the ensemble-resonator coupling can be treated in perturbation theory and the resonator dynamical equation can be solved to find the steady state phonon number

$$n_\mathrm{f}^\mathrm{LD} = \frac{\Gamma_\mathrm{c} N_0 + \gamma n_\mathrm{th}}{\Gamma_\mathrm{c} + \gamma} \tag{7}$$

where $N_0 = \mathrm{Re}\,[S(-\omega_\mathrm{m})] \,/\, \{\mathrm{Re}\,[S(\omega_\mathrm{m})] - \mathrm{Re}\,[S(-\omega_\mathrm{m})]\}$ is the minimal achievable occupancy.

To compare the error in the LD and TSD methods, we numerically solve the seven-level model explicitly, solving for the steady state using the full Hilbert space, similar to the method used in Supplementary Ref. [8]. To efficiently solve for the steady state ($\mathbf{A}x = 0$) we represent the master equation in superoperator space and explicitly construct the $\mathbf{A}$ matrix as

$$\mathbf{A}x = (-i(\mathbf{I} \otimes H - H \otimes \mathbf{I}) + \tilde{L})x, \tag{8}$$

where $x = vec(\rho)$, the vectorization of ρ, constructed by stacking the columns of ρ into a single column vector, H is the Hamiltonian of the system, $\mathbf{I}$ is the identity matrix in the total Hilbert space, and $\tilde{L}$ is the collection of Lindblad superoperators represented in superoperator space. For example, using the single Lindblad superoperator $\gamma D[a]\rho = \gamma(2a\rho a^\dagger - a^\dagger a\rho - \rho a^\dagger a)$, gives

$$\tilde{L} = \gamma(2a \otimes a - \mathbf{I} \otimes a^\dagger a - a^\dagger a \otimes \mathbf{I}). \tag{9}$$

The superoperator form of both H and the Lindblad terms are derived from the identity $\mathbf{AXB} = (\mathbf{B^T} \otimes \mathbf{A})vec(\mathbf{X})$, where $\mathbf{A}, \mathbf{B}$, and $\mathbf{X}$ are all matrices. H in Supplementary Eq. 8 can generally represent any system, but we used the seven-level Hamiltonian of Eq. 9 in the main text (or multiple instances of this, in the case of more than one NV).

$\mathbf{A}$ is an extremely large matrix, but it is also extremely sparse. For our system, $\mathbf{A}$ has less than 10 non-zeros per row, but, for the seven-level Hamiltonian, has matrix dimensions $M^2 \times M^2$, where $M = 7^N n_\mathrm{ph}$ and n_ph is the largest phonon state accessible in the simulation. We utilize the software package PETSc [9, 10] to perform these large, but very sparse,

calculations. **A** is stored in compressed sparse row format, ensuring we do the minimal amount of calculations and use the minimum amount of storage. **A** is a complex, nonsymmetric matrix, restricting us to use GMRES [11] as our parallel iterative solver, which has slow convergence, especially with increasing system size. Explicitly constructing **A** allows us to use efficient preconditioners, such as the additive Schwarz method, to accelerate the convergence. We solve for the steady state rather than doing explicit time dynamics because of the wide separation of time scales in our model. The NV dynamics are very fast, while the cooling is much slower. For an explicit time stepping approach, millions of time steps are necessary to get to the steady state solution, whereas the steady state results typically converge in less than 5000 iterations.

To understand the error from the model reduction, we first focus on simulations using just a single NV. To see significant cooling in manageable computational time, we increase the spin-strain coupling by a factor of 100 and reduce the resonator frequency to $\omega_{\mathrm{m}}/2\pi =$ 475 MHz, causing observable cooling but ensuring that the resonator is still only a small perturbation upon the NV center dynamics. We also restrict ourselves to small n_{th} values (equivalently, small temperatures) so that the Hilbert space size needed to approximate the infinite phonon bath is small and the computation remains tractable.

Supplementary Fig. 4 shows the relative error in the final phonon number n_x predicted by the TSD and the LD treatments with respect to that predicted by the seven-level simulation n_7 for one NV center. We see that, compared to LD, the TSD better approximates the numerical simulations at all values of n_{th}. As n_{th} increases, the error in the TSD results increases and then levels off at an asymptotic value of less than 0.0075, less than 1% error. At room temperature, where n_{th} is on the order of 10,000 this simplification would only give an error of only 75 phonons in the final phonon number, showing that the TSD is justified, at least for a single NV. Furthermore, it is important to note that both analytical models underestimate the cooling as compared to the numerical simulations. The LD and TSD treatments thus serve as upper bounds on the final phonon number n_{f}.

It is also important to understand how the error scales when the number of NVs is increased. This is a much more computationally challenging task, since each additional seven-level NV increases the total Hilbert space size by a factor of seven. It is only feasible to use two or three NVs, in addition to the mechanical resonator. As such, we did several calculations with one and two NVs, and a few small n_{th} values, as show in Supplementary

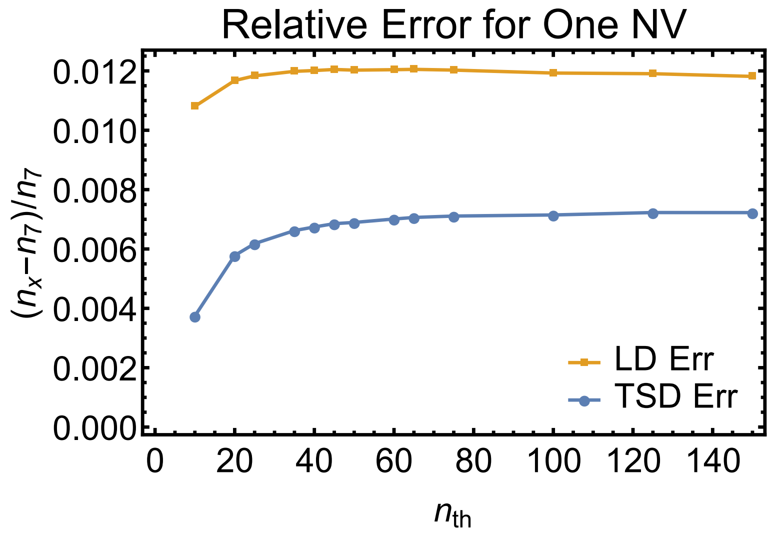

Supplementary Figure 4: Validation of two-level model for one NV center. Error in the final phonon number predicted by the analytical two-state distillation (TSD) and Lamb-Dicke (LD) treatments compared to that predicted by the numerical seven-level simulation for one NV.

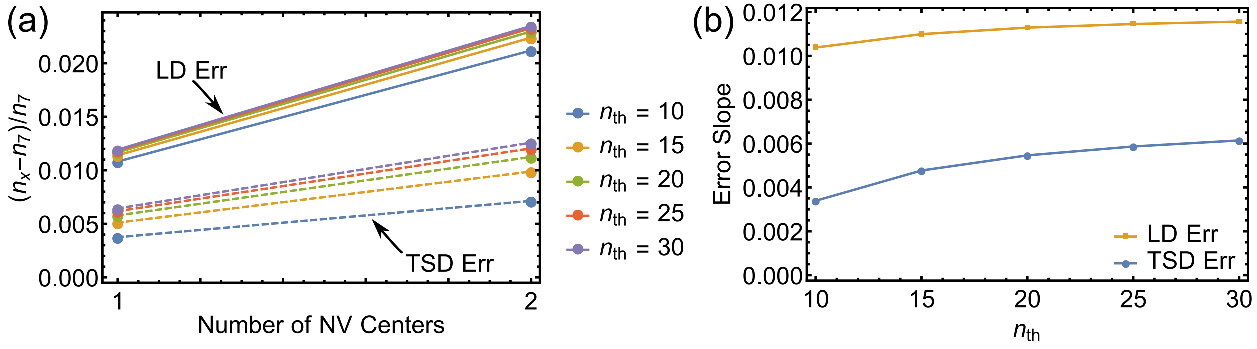

Supplementary Figure 5: Validation of two-level model for multiple NV centers. (a) Relative error of the analytical two-state distillation (TSD) and Lamb-Dicke (LD) treatments with respect to the numerical seven-level simulation for different numbers of NVs and different $n_{\rm th}$ values. (b) Slope of the lines fit to the curves in (a) plotted as a function of $n_{\rm th}$.

Fig. 5a. For both TSD and LD, the error gets worse going from one NV to two NVs, but the slope of this change is different for the different $n_{\rm th}$ values. In fact, for both treatments the slope seems to converge with increasing $n_{\rm th}$, as shown in Supplementary Fig. 5b. While it is

	$n_\mathrm{f}/n_\mathrm{th}$	
	$\nu = 2.0 \times 10^{18}$ cm^{-3}	$\nu = 4 \times 10^{20}$ cm^{-3}
Two State Distillation	0.861	0.0300
Lamb-Dicke	0.889	0.0389

Supplementary Table I: Comparison of different cooling protocol treatments.

hard to make any conclusion about the error for a dense ensemble, we can at least see that the increase in error is such that the two-level simplification is still an upper bound to the cooling, though a slightly worse one. We also did calculations with three NVs where possible, and verified that the linear behavior extends to at least three NVs. Explicitly including enough NVs to see the many NV behavior is computationally intractable, and motivates further theoretical study, such as investigations into extensions to the Tavis-Cummings model for systems with more than two states. Nevertheless, these results imply that n_f predicted by the analytical models once again serves as an upper bound, and the protocol may cool better than the models suggest.

In all cases examined here, the TSD analysis outperforms LD. Most importantly, as the number of NV centers in the ensemble grows, the TSD error grows more slowly than the LD error. This is because as λ_eff grows the spin ensemble becomes less of a perturbation on the resonator and the system departs from the LD regime. More specifically, the approximation $\lambda_\mathrm{eff}\sqrt{\langle n\rangle + 1/2} \ll 1/T_{2\mathrm{e}}^*, 1/T_{1\mathrm{e}}$ begins to fail. This failure of the LD treatment for dense ensembles at high temperature motivated our development of the TSD analysis. Nevertheless, both methods predict approximately equal cooling power for the device studied in the main text as shown in Supplementary Table I.

SUPPLEMENTARY NOTE 7: COMPARING DIFFERENT SPIN-STRAIN INTERACTIONS

The GS spin-strain interaction could also be employed to cool a mechanical resonator from room temperature. To compare the cooling efficiency of the GS interaction with that

of the ES interaction, we compute the ratio of the single-spin cooperativities

$$\begin{aligned}\frac{\eta_\mathrm{e}}{\eta_\mathrm{g}} &= \frac{\lambda_\mathrm{e}^2 T_{2\mathrm{e}}^*}{\lambda_\mathrm{g}^2 T_{2\mathrm{g}}^*} \\ &= \left(\frac{d_\perp^\mathrm{e}}{d_\perp^\mathrm{g}}\right)^2 \frac{T_{2\mathrm{e}}^*}{T_{2\mathrm{g}}^*} \\ &= (13.5)^2 \frac{T_{2\mathrm{e}}^*}{T_{2\mathrm{g}}^*}\end{aligned} \tag{10}$$

where the variables are as defined in the main text. As discussed in the main text, effects like exchange narrowing and the truncation of the spin bath make it difficult to predict $T_{2\mathrm{g}}^*$ in a nanostructure. Nevertheless, we can roughly estimate $\eta_\mathrm{e}/\eta_\mathrm{g}$ by using coherence times measured in bulk diamond.

We first treat the ensemble with an aligned NV center density $\nu = 7.0 \times 10^{17}$ cm^{-3} studied by Supplementary Ref. [4]. The GS $\{0, -1\}$ qubit coherence time for this ensemble was reported to be $T_{2\mathrm{g}}^{\{0,-1\}} = 118$ ns [4]. Because the spin-strain interaction couples the $|+1\rangle$ and $|-1\rangle$ states, the $\{+1, -1\}$ qubit coherence time sets the single-spin cooperativity. If we assume the spin dephasing is dominated by magnetic field noise, the $\{+1, -1\}$ qubit coherence of this ensemble is $T_{2\mathrm{g}}^{\{+1,-1\}} = 59$ ns. Taking $T_{2\mathrm{e}}^* = 6.0$ ns [12], the ratio of cooperativities becomes $\eta_\mathrm{e}/\eta_\mathrm{g} = 19$ as quoted in the main text.

For a more moderate density, we turn to the ensemble with $\nu = 2.8 \times 10^{13}$ cm^{-3} measured in Supplementary Ref. [1], for which $T_{2\mathrm{g}}^{\{+1,-1\}}$ was measured directly to be 450 ns [1]. This gives $\eta_\mathrm{e}/\eta_\mathrm{g} = 2.4$ as quoted in the main text.

Finally, we note that experimental demonstrations of linewidth narrowing effects in nanostructures suggest that the ES spin coherence of a dense ensemble will remain limited by the motional narrowing rate within a nanostructure [13, 14]. Our analysis of the proposed cooling protocol is thus expected to remain valid, even at very large defect densities.

SUPPLEMENTARY REFERENCES

[1] MacQuarrie, E. R. *et al.* Coherent control of a nitrogen-vacancy center spin ensemble with a diamond mechanical resonator. *Optica* **2**, 233–238 (2015).

[2] MacQuarrie, E. R., Gosavi, T. A., Jungwirth, N. R., Bhave, S. A. & Fuchs, G. D. Mechanical spin control of nitrogen-vacancy centers in diamond. *Phys. Rev. Lett.* **111**, 227602 (2013).

[3] Wilson-Rae, I., Nooshi, N., Dobrindt, J., Kippenberg, T. J. & Zwerger, W. Cavity-assisted backaction cooling of mechanical resonators. *New J. Phys.* **10**, 095007 (2008).

[4] Acosta, V. M. *et al.* Diamonds with a high density of nitrogen-vacancy centers for magnetometry applications. *Phys. Rev. B* **80**, 115202 (2009).

[5] Fuchs, G. D., Dobrovitski, V. V., Toyli, D. M., Heremans, F. J. & Awschalom, D. D. Gigahertz dynamics of a strongly driven single quantum spin. *Science* **326** (2009).

[6] Jaehne, K., Hammerer, K. & Wallquist, M. Ground-state cooling of a nanomechanical resonator via a Cooper-pair box qubit. *New Journal of Physics* **10**, 095019 (2008).

[7] Kepesidis, K. V., Bennett, S. D., Portolan, S., Lukin, M. D. & Rabl, P. Phonon cooling and lasing with nitrogen-vacancy centers in diamond. *Phys. Rev. B* **88**, 064105 (2013).

[8] Otten, M. *et al.* Entanglement of two, three, or four plasmonically coupled quantum dots. *Phys. Rev. B* **92**, 125432 (2015).

[9] Balay, S. *et al.* PETSc users manual. Tech. Rep. ANL-95/11 - Revision 3.7, Argonne National Laboratory (2016).

[10] Balay, S., Gropp, W. D., McInnes, L. C. & Smith, B. F. Efficient management of parallelism in object oriented numerical software libraries. In Arge, E., Bruaset, A. M. & Langtangen, H. P. (eds.) *Modern Software Tools in Scientific Computing*, 163–202 (Birkhäuser Press, 1997).

[11] Saad, Y. & Schultz, M. GMRES: A Generalized Minimal Residual Algorithm for Solving Nonsymmetric Linear Systems. *SIAM Journal on Scientific and Statistical Computing* **7**, 856–869 (1986).

[12] Fuchs, G. D., Falk, A. L., Dobrovitski, V. V. & Awschalom, D. D. Spin coherence during optical excitation of a single nitrogen-vacancy center in diamond. *Phys. Rev. Lett.* **108**, 157602 (2012).

[13] Smith, M. J. A., Angel, B. R. & Emmons, R. G. Distribution of substitutional nitrogen donors in synthetic diamonds. *Nature* **210**, 692–694 (1966).

[14] Baranov, P. G. *et al.* Enormously high concentrations of fluorescent nitrogen-vacancy centers fabricated by sintering of detonation nanodiamonds. *Small* **7**, 1533–1537 (2011).
